# Plasminogen activator inhibitor-1 and type 2 diabetes: a systematic review and meta-analysis of observational studies

**DOI:** 10.1038/srep17714

**Published:** 2016-01-27

**Authors:** James Yarmolinsky, Natália Bordin Barbieri, Tobias Weinmann, Patricia K. Ziegelmann, Bruce B. Duncan, Maria Inês Schmidt

**Affiliations:** 1 Postgraduate Program in Epidemiology, Faculty of Medicine, Federal University of Rio Grande do Sul, Porto Alegre, RS, Brazil; 2 Occupational and Environmental Epidemiology & NetTeaching Unit, Institute and Outpatient Clinic for Occupational, Social and Environmental Medicine, University Hospital of Munich, Ludwig Maximilian University of Munich, Munich, Germany; 3 Department of Statistics, Federal University of Rio Grande do Sul, Porto Alegre, RS, Brazil

## Abstract

An emerging body of evidence has implicated plasminogen activator inhibitor-1 (PAI-1) in the development of type 2 diabetes (T2D), though findings have not always been consistent. We systematically reviewed epidemiological studies examining the association of PAI-1 with T2D. EMBASE, PubMed, Web of Science, and the Cochrane Library were searched to identify studies for inclusion. Fifty-two studies (44 cross-sectional with 47 unique analytical comparisons and 8 prospective) were included. In pooled random-effects analyses of prospective studies, a comparison of the top third vs. bottom third of baseline PAI-1 values generated a RR of T2D of 1.67 (95% CI 1.28–2.18) with moderate heterogeneity (I^2^ = 38%). Additionally, of 47 cross-sectional comparisons, 34(72%) reported significantly elevated PAI-1 among diabetes cases versus controls, 2(4%) reported significantly elevated PAI-1 among controls, and 11(24%) reported null effects. Results from pooled analyses of prospective studies did not differ substantially by study design, length of follow-up, adjustment for various putative confounding factors, or study quality, and were robust to sensitivity analyses. Findings from this systematic review of the available epidemiological literature support a link between PAI-1 and T2D, independent of established diabetes risk factors. Given the moderate size of the association and heterogeneity across studies, future prospective studies are warranted.

Procoagulant and fibrinolytic markers have been proposed as risk factors for the development of type 2 diabetes^1^. Plasminogen activator inhibitor-1 (PAI-1), a serine-protease inhibitor secreted primarily by adipocytes, endothelial cells, and hepatocytes, acts as a key negative regulator of fibrinolysis through its role as the primary inhibitor of tissue plasminogen activator (tPA). Experimental studies in mice homozygous for the PAI-1 null allele have found favourable effects on insulin and glycaemic measures^2^ and protective effects against the development of obesity and insulin resistance when fed a high-fat/high-carbohydrate diet^3^, as compared with wild-type mice. Likewise, early cross-sectional studies in humans have reported associations of elevated PAI-1 concentrations with measures of obesity^4^,^5^, insulin resistance^4^,^6^, impaired glucose tolerance (IGT)^4^,^6^, and T2D^7^,^8^. These findings have been extended to a prospective context by investigators of the Insulin Resistance Atherosclerosis Study (IRAS) who reported that elevated PAI-1 levels were an independent risk factor for the development of T2D in healthy subjects, after 5.2 years of follow-up^9^.

Since the publication of these initial studies, a considerable number of additional observational studies have been published, with many, but not all, reporting associations of PAI-1 with T2D^10^,^11^,^12^,^13^,^14^,^15^,^16^,^17^. To our knowledge, however, no attempt has been made to consolidate and synthesize the available epidemiological literature on this topic in the form of a systematic review and meta-analysis. Thus, in light of the heterogeneity of findings and the need to quantify the relationship of PAI-1 with diabetes, we performed a systematic review and meta-analysis of observational studies examining the association between plasminogen activator inhibitor-1 and type 2 diabetes.

## Methods

### Literature Search

We conducted a comprehensive literature search of the bibliographic databases EMBASE, PubMed, Web of Science, and the Cochrane Library for all relevant studies, published from 1945 to October 2014. Medical subject headings (MeSH) or equivalent and text word terms were utilised. Search strategies were individualised to specific databases and are presented for each database in Supplementary Data 1. The study protocol is registered with the PROSPERO database of systematic reviews (http://www.crd.york.ac.uk; registration number CRD42014014009).

Titles and abstracts were screened by two independent reviewers (JY,NBB) for inclusion according to pre-specified criteria (see below). If an abstract was not available for a study, the full article was obtained and screened. If an article appeared to be potentially eligible for inclusion based on title and/or abstract, the full article was obtained and formally screened for inclusion, otherwise it was excluded. When duplicate analyses appeared to be presented across more than one publication, we included only the first publication. Reference lists for included studies were screened for additional relevant studies. Lastly, corresponding authors were contacted for additional information pertinent to study inclusion if necessary.

### Inclusion and exclusion criteria

Included studies had to meet all of the following inclusion criteria: 1) prospective or retrospective cohort, case-cohort, case-control, or cross-sectional study; 2) Measurement of plasma PAI-1 (antigen concentrations or activity levels); 3) Assessment of T2D (self-reported physician diagnosis and/or medication usage and/or laboratory diagnosed); 4) Adult study population (≥18 years) at baseline; 5) Article was reported in English. In epidemiological studies of the association of plasma PAI-1 with T2D, PAI-1 is typically measured using either an assay that is sensitive to free PAI-1 antigen (both active and latent forms) that is not complexed to plasminogen activators or an assay that detects activity level (active free PAI-1). Both free PAI-1 antigen and activity levels have been shown to strongly correlate with each other^17^. Thus, studies that examined plasma PAI-1 as antigen or as activity level were both included in this review and pooled in the meta-analysis of prospective studies. We excluded all animal studies, case reports, and editorials. Studies were further excluded if they provided outcome data solely on gestational diabetes or type 1 diabetes.

### Data extraction and Quality Assessment

Using a standardized data extraction form, two independent reviewers (JY,TW) extracted relevant information from each paper and this information was reported in accordance with guidelines established by the Meta-analysis of Observational Studies in Epidemiology (MOOSE) checklist^18^. Any discrepancies between reviewers were reconciled by consensus. The following information (if available) was extracted from each study: authors, year of publication, country of origin, study design, sample demographic characteristics, number of cases and controls, covariates adjusted or “matched” for, mean or median PAI-1 concentrations of cases and controls for cross-sectional studies, and for prospective studies: duration of follow-up, method of incident T2D assessment, assay method, effect estimates with 95% CIs, and data pertinent to methodological quality assessment. If sex-specific analyses were presented, numbers of cases and controls and effect estimates from both sexes were extracted. For cross-sectional studies, if PAI-1 values for T2D groups with and without co-morbidities were presented, information from the group with no or minimal co-morbidities was extracted. If a study presented PAI-1 data for both antigen concentration and activity level, only PAI-1 antigen levels were extracted. Authors were contacted for additional information if any required data from a study was missing or unclear. Study quality was independently assessed by two reviewers (JY,NBB) using the Newcastle-Ottawa Quality Assessment Scale (NOS) to examine the selection of participants and study design, comparability of groups, and ascertainment of exposure/outcome^19^. Scores for low (0–3), moderate (4–6), and high-quality studies (7–9) were assigned.

### Statistical analyses

For prospective studies, relative risks (RRs) were used to measure the association between PAI-1 levels and incident T2D. Hazard ratios (HRs) and odds ratios (ORs) were assumed to approximate the same measure of RR. In studies where associations between PAI-1 and incident T2D were presented through multiple sequentially-adjusted models, the effect estimate and 95% CIs from the most fully-adjusted model was extracted and used in the meta-analysis. In order to permit comparison of effect between studies that used different categorical or continuous comparisons, a scaling factor was employed to convert reported RRs of seven studies to upper and lower tertile comparisons, using methods previously reported^20^. These scaling methods assume a log-normal distribution of baseline PAI-1 concentrations and a log-linear association with diabetes risk. Briefly, effect estimates from four studies that employed a 1-SD comparison of baseline PAI-1 were multiplied by a scaling factor of 2.18 (the equivalent of the difference between means in the top and bottom third of the distribution)^9^,^12^,^21^,^22^, the effect estimate from one study that employed an interquartile range comparison was scaled by 1.61 (2.18/1.35; difference in means between top and bottom third of distribution)^23^, one study that employed a quartile comparison was scaled by 0.858 (2.18/2.54; difference in means between top and bottom quartile)^24^, and one study that compared unequal groups was multiplied by a study-specific scaling factor of 2.18/*x* where *x* is the difference in mean PAI-1 (in SD units) between the two groups^10^. Standard errors of the log RRs were calculated using published confidence limits and standardised in the same manner. Studies were weighted by the inverse of the variance of each transformed log RR and then pooled to generate estimates of average effect using random-effects models. The I^2^ statistic was used to determine the percentage of variability across studies due to heterogeneity beyond chance and its significance tested using the Cochran Q test. For cross-sectional studies, owing to considerable methodological heterogeneity and the lack of standardized PAI-1 measurement protocols, a meta-analysis of the data was not considered appropriate. Consequently, data from cross-sectional studies were summarized in aggregate.

Sources of heterogeneity among prospective studies were examined by comparing analyses stratified by study design (prospective cohort vs. nested case-control), duration of follow-up (median split), baseline glucose tolerance status (normal glucose tolerance vs. glucose intolerance [IFG or IGT]), adjustment for glucose, adjustment for insulin measures (HOMA-IR, fasting insulin, insulin sensitivity index [S_I_]), adjustment for visceral adiposity (waist-hip ratio [WHR] or waist circumference [WC]), and adjustment for inflammatory markers. Additionally, a meta-regression was performed by modelling duration of follow-up as a continuous variable with T2D risk. Sensitivity analyses were performed by comparing pooled risk estimates to those generated after: iteratively removing one study at a time to confirm that our findings were not driven by any single study, removal of studies with a NOS score <7, and employing a fixed-effects model. The presence of publication bias in the meta-analysis was assessed using tests of asymmetry (Begg’s, Egger) and visual inspection of funnel plots^25^,^26^. All statistical tests were two-sided and significance was defined at *p*< 0.05. Statistical analyses were performed with R version 3.2.1 and Revman 5.2 (Nordic Cochrane Center).

## Results

### Literature Search

**Figure 1:**
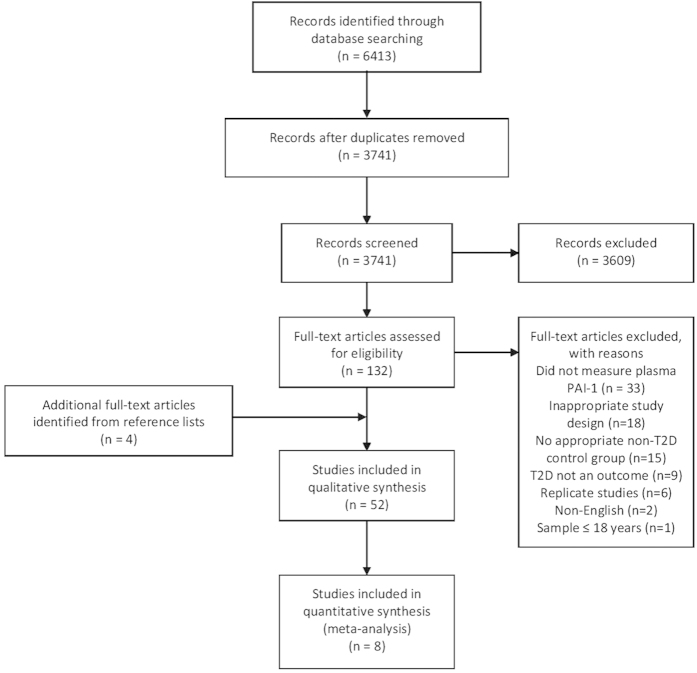
Modified PRISMA flow diagram through study search and inclusion.

Our search strategy initially identified 6413 articles, which was reduced to 3741 unique citations after removal of duplicate studies. From these studies, 3609 were excluded based on title and/or abstract, and 84 were further excluded after retrieval of full-text articles. Screening of reference lists of included studies identified an additional 4 studies for inclusion. Consequently, a total of 52 studies (44 cross-sectional with 47 unique analytical comparisons and 8 prospective) were included in this review [Fig. 1].

### Study Characteristics and Statistical Analyses

#### Cross-Sectional Studies

Descriptive characteristics of the 44 cross-sectional studies are presented in Table 1^9^,^10^,^12^,^21^,^22^,^23^,^24^,^27^. In brief, 23 studies were conducted in Europe, 12 in Asia, 5 in the U.S.A, 2 in South America, 1 in the Middle East, and 1 in Africa. 21 studies reported adjustment for some covariates (mainly age and sex), while 23 did not report adjustment for covariates. 35 studies enrolled both men and women as participants, 4 enrolled exclusively one sex, and in 5 studies it was unclear. 30 studies assessed PAI-1 in its antigen form and 14 as activity level.

Overall, 34 out of 47 analytical comparisons (72%) reported statistically significant elevated mean or median PAI-1 (antigen or activity) among T2D cases as compared to controls, 2 (4%) reported statistically significant elevated mean PAI-1 antigen among controls versus cases, and 11 (24%) reported no association of PAI-1 with outcome status. 16 of 23 (70%) comparisons that reported some form of statistical adjustment for covariates reported significantly elevated PAI-1 among diabetes cases, as compared to 18 of 24 (75%) that did not report any adjustment. 8 out of 13 (62%) comparisons from Asia reported significant elevated PAI-1 among diabetes cases, whereas 23 out of 30 (77%) from Europe or North America found elevated PAI-1 among cases. 4 out of 5 comparisons exclusively between women reported significantly elevated PAI-1 among cases, whereas 2 out of 5 between men reported elevated PAI-1 among cases.

#### Prospective Studies

Eight prospective studies, including a total of 9256 participants and 980 incident T2D cases, were included in this review [Table 2]^9^,^10^,^12^,^21^,^22^,^23^,^24^,^27^. Five studies were conducted in the U.S.A.^9^,^12^,^23^,^24^,^27^, two in Sweden^10^,^22^, and one in France^21^ and all included both men and women as participants. Five studies were prospective cohort analyses^9^,^10^,^12^,^23^,^24^ and three were nested case-control studies^21^,^22^,^27^. Six studies examined baseline PAI-1 in its antigen form^9^,^12^,^22^,^23^,^24^,^27^ and two as activity level^10^,^21^. Length of follow-up ranged from four to nine years with a median length of 5.7 years. Incident diabetes was ascertained using a combination of fasting glucose ≥7.0 mmol/L (or ≥126 mg/dL) and self-report of diabetes diagnosis or diabetes medication use in five studies^10^,^12^,^21^,^22^,^23^,^24^,^27^. In two studies, postload glucose ≥11.1 mmol was included as an additional criterion^10^,^22^. In one study, diabetes status was ascertained solely with a standard 75-g OGTT^9^. Most studies reported adjustment in their models for established diabetes risk factors including age, sex, triglyceride levels, BP or hypertension, measures of insulin or insulin resistance, and measures of overall or visceral adiposity. Lastly, in quality assessment of these studies, seven were categorised as being of “high” methodological quality (NOS score > 6) and one as “moderate” quality (NOS score =6) [Table t1][Table t2][Tables 3, 4].

**Figure 2:**
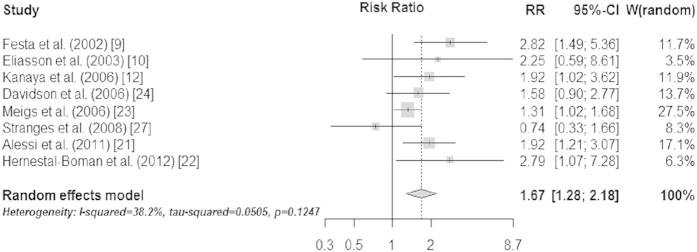
Individual and pooled risk ratios and 95% confidence intervals for random-effects model examining the association between the top vs. bottom third of baseline plasminogen activator inhibitor-1 levels and type 2 diabetes.

When the eight prospective studies were pooled in a random-effects meta-analysis, a comparison of the top third vs. bottom third of baseline plasminogen activator inhibitor-1 concentrations generated a summary relative risk of T2D of 1.67 (95% CI 1.28–2.18) [Fig. 2]. There was moderate evidence for heterogeneity across studies (I^2^=38.2%, *p*=0.12), mostly accounted for by Stranges *et al.* which reported a non-significant “protective” association of elevated PAI-1 with diabetes risk (removal of this study from the meta-analysis reduced the I^2^ statistic to 25% (*p*=0.24) without materially changing the pooled RR [1.74, 95% CI 1.37–2.22])^27^.

**Figure 3:**
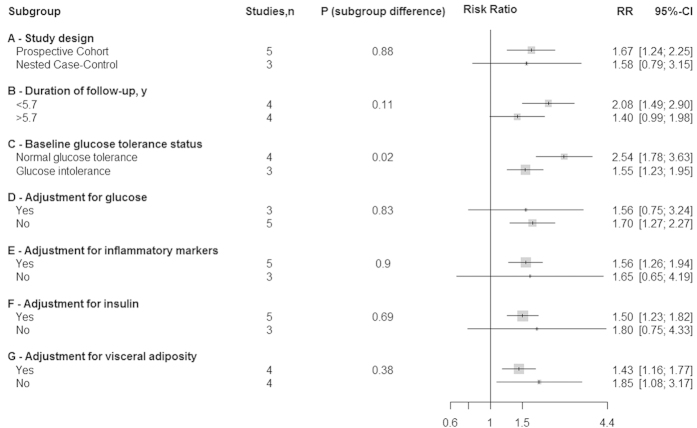
Individual and pooled risk ratios and 95% confidence intervals for random-effects model examining the association between the top vs. bottom third of baseline plasminogen activator inhibitor-1 levels and type 2 diabetes, by sub-group analysis.

Studies that presented results stratified by baseline glucose tolerance status or limited participants exclusively to those with normal glucose tolerance at baseline suggested an increased risk of diabetes, in relative terms, among participants with normal glucose tolerance at baseline compared to those with glucose intolerance (normal glucose tolerance: RR 2.54, 95% CI 1.78–3.63; glucose intolerance: RR 1.55, 95% CI 1.23–1.95; *p*=0.02 for sub-group difference), though these analyses were limited to seven comparisons from four studies^9^,^10^,^22^,^23^ [Fig. 3]. Further, a sensitivity analysis performed by removal of Meigs *et al.* from both sub-groups attenuated these differences toward non-significance suggesting substantial influence of this study on sub-group differences (normal glucose tolerance: RR 4.00, 95% CI 1.95–8.23; glucose intolerance: RR 2.21, 95% CI 1.14–4.27; *p*=0.23 for sub-group difference). Additionally, in secondary sub-group analyses comparing studies by adjustment for BMI, which were not specified a priori, we found a stronger association in studies that adjusted for BMI as compared to those that did not (adjustment for BMI: RR 2.18, 95% CI 1.60–2.96; no adjustment for BMI: RR 1.31, 95% CI 1.06–1.93; *p*-sub-group difference=0.008). However, as in sensitivity analyses performed by baseline glycemia status, removal of Meigs *et al.* from this sub-group analysis attenuated these differences toward non-significance (adjustment for BMI: RR 2.18, 95% CI 1.60–2.96; no adjustment for BMI: RR 1.29, 95% CI 0.73–2.31; *p*-sub-group difference=0.12). No significant sub-group differences were found for all other sub-group analyses though statistical power may have been limited to detect any differences present [Fig. 3]. Additionally, we failed to find a significant linear association of duration of follow-up (in years) with T2D risk in a meta-regression (*p*=0.94). Although it was our intention to look into potential differential associations of PAI-1 with diabetes by sex, the lack of studies reporting stratified analyses by sex prevented this.

**Figure 4:**
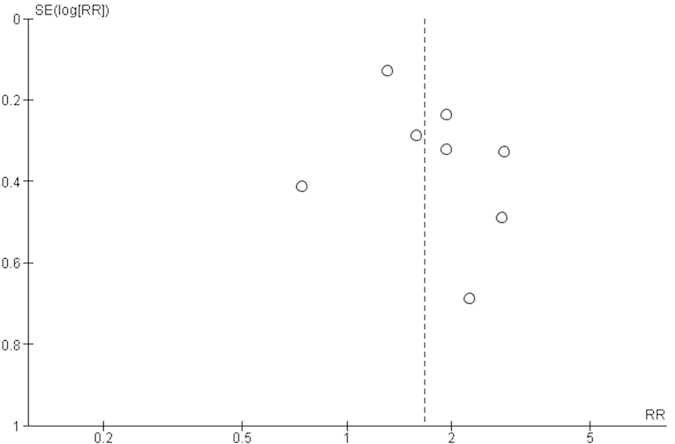
Funnel plot for 8 prospective studies examining the association of plasminogen activator inhibitor-1 levels with risk of type 2 diabetes.

Pooled effects were robust to sensitivity analyses performed by removal of one study with “moderate” methodological quality (NOS score: 6) from our pooled analysis, use of the “leave-one-out” method, and use of a fixed-effects model. Visual inspection of funnel plots did not reveal substantial asymmetry indicative of small-study bias [Fig. 4]. Results from Begg’s (*p*=0.46) and Egger’s (*p*=0.26) tests further suggested absence of publication bias.

## Discussion

This systematic review of 52 epidemiological studies supports a link between plasminogen activator inhibitor-1 and type 2 diabetes, thus highlighting a potentially significant yet under-appreciated risk factor for diabetes. While cross-sectional studies were not aggregated in a meta-analysis, most studies reported significantly elevated PAI-1 levels among individuals with T2D, as compared to controls. Pooled analyses of 8 prospective studies revealed a 67% increased risk of T2D (upper vs lower tertiles of baseline PAI-1) at a median follow-up of 5.7 years. Notably, this association was maintained in analyses adjusted for established risk factors for diabetes, including various measures of the metabolic syndrome. In sub-group analyses, greater risk of T2D was reported for participants with normal glucose tolerance at baseline, as compared to those with glucose intolerance, though this difference was no longer statistically significant after removal of the largest study. Sensitivity analyses performed by removal of one “moderate” methodological quality study, use of the “leave-one-out” method, and use of fixed-effects models did not substantially modify associations, thus supporting the robustness of our overall pooled effect estimate. Lastly, though relatively few studies contributed to our meta-analysis of prospective studies, we did not find evidence for publication bias.

The primary rationale for performing this systematic review and meta-analysis was to provide the first comprehensive summary of the available epidemiological literature on the association of PAI-1 with T2D, which we consider to be the primary strength of this review. Pooled prospective studies adjusted for a comprehensive panel of established diabetes risk factors, allowing for investigation of independent effects of PAI-1 on diabetes risk, though the presence of residual or unknown confounding from individual studies cannot be ruled out. Finally, in quality assessment of the prospective studies included in our review, seven studies were considered to be of “high” methodological quality with the remaining one being considered “moderate” methodological quality.

Some limitations of this review deserve mention. Firstly, it is important to emphasize the inherent limitation in inferring causal direction of associations from cross-sectional studies. While pooled analyses of prospective studies suggest that heightened levels of PAI-1 can predict development of T2D, we cannot rule out a potential aetiological role of the diabetic state in further influencing PAI-1 levels. Secondly, approximately half of the cross-sectional studies included in our review did not report any adjustment for the presence of possible confounding factors. However, the lack of a substantial difference in the proportion of unadjusted and adjusted cross-sectional studies that reported elevated PAI-1 levels among those with diabetes and the maintenance of an elevated risk of diabetes in pooled adjusted analyses of prospective studies would not appear to suggest a strong role of known confounding influencing these results. Thirdly, while a comprehensive literature search was performed to identify all observational studies examining an association of PAI-1 with T2D, only eight prospective studies were identified and included in our meta-analysis. Consequently, the total number of incident cases in our meta-analysis was relatively small and statistical power was limited in sub-group analyses and tests for publication bias. Additionally, prospective studies were confined to American and European populations, potentially limiting external validity of findings to other genetically-distinct populations. This issue is particularly relevant in light of evidence suggesting significant differences in circulating PAI-1 levels between various ethnic groups^28^,^29^,^30^ in addition to the differences between these groups in both susceptibility to T2D^31^ and in the relative contribution of established risk factors to diabetes development^32^. Lastly, we found moderate heterogeneity across studies, although a substantial contribution to this heterogeneity was provided by a single study which reported a “protective” association of elevated PAI-1 with diabetes risk. It is feasible that the relatively small size of this study may have contributed to a chance finding.

That other circulating markers of endothelial dysfunction such as coagulation factor VIII, E-selectin, intercellular adhesion molecule-1, and tPA have also been found to predict T2D^10^,^33^,^34^,^35^,^36^,^37^, also supports a role of PAI-1 in the pathogenesis of T2D. Of particular relevance are three studies that have reported associations of tPA activity and tPA antigen with risk of diabetes^10^,^22^,^35^, as PAI-1 serves as the primary regulator of tPA in the fibrinolytic pathway and the PAI-1/tPA antigens have been shown to be highly correlated^38^. Further, in a follow-up of their initial analysis in the IRAS, Festa *et al.* reported that change in PAI-1 over time, in addition to elevated baseline levels, predicted incident diabetes^39^. Additionally, alleles of various SNPs which elevate plasma PAI-1 have been found to be individually associated with an increased odds of T2D^40^. Lastly, successful randomised, controlled diabetes prevention trials involving lifestyle and pharmacological interventions have been shown to decrease plasma PAI-1 levels^41^,^42^,^43^.

Various putative mechanisms have been proposed to explain the association of PAI-1 with T2D. Animal models have suggested that PAI-1 may play a causal role in the development of obesity and insulin resistance^2^,^3^ and elevated PAI-1 in humans has been shown to predict incident metabolic syndrome in two prospective studies^21^,^44^.

The majority of circulating PAI-1 is synthesized by adipose tissue^45^. Visceral fat has been shown to secrete more PAI-1 than subcutaneous fat tissue in the obese phenotype^46^,^47^,^48^ and WHR has been found to correlate more strongly with PAI-1 than BMI^49^,^50^,^51^. Lifestyle and dietary-mediated weight loss in the moderately overweight and obese has been associated with concomitant reductions in PAI-1 levels, further supporting a causal link between adiposity and PAI-1 expression^52^,^53^,^54^,^55^. However, in our review, 7 of the prospective studies that adjusted for measures of overall (BMI) or visceral adiposity (WHR or WC) maintained a significant association between PAI-1 and diabetes, though the association was attenuated in some studies. Further, when we stratified our analyses by studies with adjustment for WHR or WC, we failed to find substantial heterogeneity in associations across studies that did and did not control for measures of visceral adiposity.

Adipose tissue is also responsible for the secretion of various pro-inflammatory cytokines, markers of low-grade chronic inflammation that have been linked with development of insulin resistance^56^. Further, these adipocytokines have all been shown to up-regulate production of PAI-1^57^,^58^,^59^,^60^,^61^,^62^. Thus, the association of PAI-1 with incident T2D could simply reflect residual confounding of an association of one or more other inflammatory markers with diabetes. In our meta-analysis, four of the prospective studies that adjusted for C-reactive protein (CRP) did not report strongly attenuated associations after including this inflammatory marker in their models. Further, Kanaya *et al.* reported the maintenance of an association of PAI-1 with diabetes after adjusting their model for baseline concentrations of leptin and adiponectin, two other adipocytokines involved in regulating inflammatory tone which have been found to modulate T2D risk^12^,^63^,^64^. Likewise, we failed to find evidence of heterogeneous associations of PAI-1 with diabetes across studies stratified by adjustment for any inflammatory markers.

Though PAI-1 has been demonstrated to contribute to insulin resistance, insulin has also been shown to stimulate PAI-1 secretion by fat cells, in a pathway that is upregulated in hyperinsulinemia and hyperglycemia^65^,^66^, thus suggesting that the relationship between endothelial dysfunction and insulin resistance is bi-directional^67^. In our review, an initial association of elevated PAI-1 with risk of T2D in Davidson *et al.* became non-significant upon adjustment for baseline insulin measures^24^. Similarly, an initial increased risk of T2D with elevated PAI-1 activity in Eliasson *et al.* was no longer significant after adjustment for fasting insulin, diastolic blood pressure, and triglycerides, though this may have led to over-parameterization in their model owing to the limited number of incident diabetes cases in the study (n=15)^10^. In contrast, the three other prospective studies in our review that adjusted for insulin reported maintenance of their respective associations. When we compared associations of PAI-1 with diabetes across studies adjusting for measures of insulin (fasting insulin, HOMA-IR, S_I_), we failed to find evidence of differential risk across sub-groups.

Lastly, while elevated PAI-1 levels have been shown to predict incident hyperglycemia, plasma glucose levels may also influence PAI-1 secretion. For example, experimental studies in both animals and humans have shown that glucose up-regulates PAI-1 gene expression in vascular smooth muscle cells, endothelial cells, and adipose tissue^68^,^69^,^70^,^71^. Thus, it could be expected that as glucose levels rise, enhanced PAI-1 gene expression would result in elevated circulating PAI-1 concentrations. However, in sub-group analyses performed on studies stratified by adjustment for glucose we failed to find evidence for heterogeneous associations across studies that did and did not include glucose measures in their models. On the contrary, results from our sub-group analyses of studies stratified by baseline glucose tolerance status of participants suggested a significantly greater risk of T2D with heightened PAI-1 levels among participants with normal, as compared to elevated glucose levels, at baseline, though this difference appeared to be driven primarily by one study.

Recently, a novel explanation for the relationship between PAI-1 and diabetes was provided by Lee *et al.* who examined the association of a group of inflammatory markers with longitudinal changes in the Metabolic Clearance Rate of Insulin (MCR) in the Insulin Resistance Atherosclerosis Study^72^. It was reported that among 784 non-diabetic participants, higher baseline levels of plasma PAI-1, but not of CRP, TNF-α, leptin, or fibrinogen, were associated with a decline in the MCR after five years of follow-up. This association remained significant after adjustment for measures of adiposity, fasting blood glucose, insulin sensitivity, and CRP.

Taken together, the available literature appears to support a role of PAI-1 as both contributor to and consequence of the metabolic syndrome. However, our meta-analysis of eight prospective studies found an association of PAI-1 with T2D risk in multivariate analyses adjusting for various measures of the metabolic syndrome including central adiposity, insulin resistance, and hyperglycemia, in addition to other established risk factors for diabetes. Consequently, this independent association could conceivably support a pathophysiological mechanism distinct from the metabolic syndrome.

Thus, these findings, by furthering the understanding of the causal pathways involved in diabetes pathophysiology, could potentially help shape future prevention strategies including pharmacological interventions. Further, investigation of the addition of PAI-1 to diabetes risk prediction models could provide insight into the clinical utility of inclusion of PAI-1 into diabetes screening tools. Findings from this review additionally extend evidence of a role of elevated PAI-1 in diabetic retinopathy^73^, diabetic nephropathy^74^, and coronary heart disease in individuals with type 2 diabetes^74^, to the risk of incident diabetes, thus identifying a role of PAI-1 in contributing to morbidity across the blood glucose continuum. In light of the relative homogeneity of the sample populations included in the eight prospective studies in our review and the identification of significant heterogeneity between ethnic groups in circulating PAI-1 levels, further prospective examination of the association of PAI-1 with diabetes in non-Caucasian samples is warranted. Additionally, findings from our review suggest the need for investigation of the role of PAI-1 on diabetes risk within cohorts that can permit greater exploration of the potential role of differences in baseline glycaemia, on this association. Our findings also identify a potential role for randomised controlled trials to determine the effectiveness of pharmacological interventions targeting impaired fibrinolysis in order to prevent new cases of T2D. Lastly, with the recent identification of a number of functionally relevant SNPs for circulating PAI-1 concentrations^40^,^75^, Mendelian randomisation analyses could provide an additional tool for exploration of a causal effect of PAI-1 on diabetes risk.

## Additional Information

**How to cite this article**: Yarmolinsky, J. *et al.* Plasminogen activator inhibitor-1 and type 2 diabetes: a systematic review and meta-analysis of observational studies. *Sci. Rep.* **6**, 17714; doi: 10.1038/srep17714 (2016).

## Supplementary Material

Supplementary Information

## Figures and Tables

**Table 1 t1:** Characteristics of cross-sectional studies of plasminogen activator inhibitor-1 and type 2 diabetes.

Study	Country	No. of ases/controls	Mean age (SD); % Women; Ethnicity	Adjustments	Mean or median PAI-1, ng/mL^a^		*P*-value
					Cases	Controls	
Auwerx (1988)^76^	Belgium	33/57	63.1 (9.4) for cases, 52.0 (12.0) for controls; 45.6%; European	None	*3.3*	*1.5*	<0.001
Juhan-Vague (1989)^77^	France	38/20	57.6 (12.0) for cases, 52.3 (9.1) for controls; 53.4%; European	Age	*21.8*	*7.7*	<0.001
Rydzewski (1990)^78^	Japan	31/20	55.9 (11.9) for cases, 49.4 (20.8) for controls; 29%; Asian	None	8.7	7.2	≥0.05
Cho (1992)^79^	South Korea	49/16	51.3 (14.9) for cases, 49.8 (12.2) for controls; 51%; Asian	Age	35.9	17.6	<0.05
Potter van Loon (1993)^80^	Netherlands	10/9	52 (2) for cases, 47 (2) for controls; 42%; European	None	44	34	<0.01
Takada (1993)^81^	Japan	43/95	53.9 (12.7) for cases, N/A for controls; 0%; Asian	None	5.9	15.0	<0.001
Avellone (1994)^82^	Italy	22/20	48.5 (2.5) for cases, 47.5 (3.0) for controls; 60%; European	Sex, age, BMI	*6.6*	*1.4*	<0.01
Park (1994)^83^	South Korea	64/32	56.1 (9.5) for cases, 57.9 (8.9) for controls; 62.5%; Asian	Sex, age, BMI, WHR	27.7	27.7	≥0.05
Kario 1995^84^	Japan	31/42	67 (9.9) for cases, 67 (6.6) for controls; 55%; Asian	Sex, age, BMI	7.9	4.2	<0.01
Ito (1996)^85^	Japan	77/10	59.1 (1.2) for cases, 40.0 (2.7) for controls; 44.8%; Asian	None	9.3	4.3	<0.05
Nagi (1996)^8^	U.K.	84/149	55.0 (8.0) for cases, 49.0 (8.0) for controls; 41.6%; Asian and Caucasian European	Sex, age	*23.0*	*17.1*	<0.001
Akanji (1997)^86^	Kuwait	32/68	47.8 (7.4) for cases, 39.1 (11.0) for controls; N/A; Middle eastern	None	43.8	32.5	<0.01
Gray (1997)^87^	U.K.	30/38	60.2 (9.0) for cases, 57.5 (8.1) for controls; 0%; European	None	*11.2*	*10.6*	≥0.05
Krekora (1997)^88^	Italy	59/50	63.0 (10.0) for cases, 59.8 (7.1) for controls; 28.4%; European	Age	107	29.1	<0.001
Bannan (1998)^89^	U.K.	60/60	60.3 (9.5) for cases, 44.5 (13.0) for controls; 50.0%; European	Sex, age, BMI,	*22.6*	*10.5*	0.00001
Hughes (1998)^17^	Singapore	Men: 72/248; Women: 54/282	40–69 years; 51%; Asian	Age, ethnicity	Men: 28.6 Women: 32.4	Men: 23.9 Women: 24.6	Men: 0.06 Women:<0.01
Temelkova-Kurktschiev (1999)^90^	Germany	68/249	56.4 (0.9) for cases, 53.5 (0.5) for controls; 59.3%; European	None	79.2	57.6	<0.05
Testa (1998)^91^	Italy	66/31	62.7 (11.7) for cases, 57.3 (12.7) for controls; 33.0%; European	None	29.0	29.1	≥0.05
Festa (1999)^4^	U.S.A.	510/693	57.3 (0.4) for cases, 53.9 (0.3) for controls; 53.4%; Non-Hispanic white, African-American, Mexican-American	Sex, age, ethnic group, clinic, BMI, insulin sensitivity	28.9	23.0	<0.001
Testa (1999)^92^	Italy	49/87	62.2 (10.1) for cases, 64.1 (8.7) for controls; N/A; European	None	29.4	30.8	≥0.05
Hernandez (2000)^93^	Spain	41/40	59.8 (10.3) for cases, 43.0 (14.2) for controls; 60%; European	None	51.3	23	<0.05
Testa (2000)^94^	Italy	73/46	61.5 (10.5) for cases, 63.1 (12.7) for controls; 47.9%; European	None	32.8	30.1	≥0.05
Zareba (2001)^95^	U.S.A.	125/846	62.0 (11.0) for cases, 59.0 (12.0) for controls; 22.9%; White, black	None	*38*	*27*	<0.017
Aso (2002)^96^	Japan	112/69	57.9 (10.9) for cases, 54.5 (12.1) for controls; 49%; Asian	Age	12.3	9.0	<0.05
Brandenburg (2002)^97^	U.S.A.	Men: 8/8 Women: 8/8	42.0 (6.8) for cases, 39.0 (7.1) for controls; 50%; North American	None	Men: *27.7* Women: *35.1*	Men: *25.4* Women: *24.0*	Men: ≥0.05 Women: ≥0.05
Leurs (2002)^98^	Netherlands	47/51	69.0 (8.0) for cases, 65.0 (6.0) for controls; 52.0%; European	Age	*18*	*13*	<0.001
Fattah (2004)^99^	Egypt	15/15	41–65 years; N/A; North African	Sex, age	*4.3*	*2.0*	0.001
Kanaya (2004)^11^	U.S.A.	Men: 298/298 Women: 221/221	73.7 (2.9) for cases, 73.5 (2.9) for controls; 42.6%; White, black	Sex, race, study site	Men: 26 Women: 33	Men: 17 Women: 20	Men: <0.001 Women:<0.001
Yu (2004)^100^	China	12/12	59.0 (3.0) for cases, 48.0 (8.0) for controls; 50.0%; Asian	None	45.4	33.6	≥0.05
Erem (2005)^101^	Turkey	92/40	50.1 (13.4) for cases, 49.8 (15.1) for controls; 52%; European	Sex, age, BMI	44.6	21.4	<0.0001
Kitagawa (2006)^16^	Japan	47/31	53.4 (13.6) for cases, 52.3 (11.5) for controls; 41.0%; Asian	Age	82.7	52.9	<0.05
Soares (2007)^102^	Brazil	7/16	52.1 (8.3) for cases, 52.3 (5.4) for controls; 52%; Latin American	None	70.5	27.5	0.03
Le (2008)^103^	U.S.A.	104/59	32.0 (4.0) for cases and controls; 60.1%; Indian American	Age	39	31	≥0.05
Romuk (2008)^104^	Poland	20/21	61.1 (8.4) for cases, 47.9 (7.1) for controls; N/A; European	None	*10.6*	*3.9*	<0.0001
Sahli (2009)^105^	Sweden	55/73	52 (9) for cases, 48 (11) for controls; 48%; European	None	*39.7*	*10.5*	<0.0001
Jax (2009)^106^	Germany	26/122	57(4) for cases, 58(5) for controls; N/A; European	None	*7.4*	*5.5*	0.0017
Blaszkowski (2010)^107^	Poland	53/24	N/A; 47.2% for cases, N/A for controls; European	Sex, age	55.3	27.7	<0.0001
Kubisz (2010)^108^	Slovakia	42/42	61.8 (7.8) for cases, 55.4 (6.0) for controls; 54%; European	None	72.0	27.9	<0.0001
Kovalyova (2011)^109^	Ukraine	24/51	N/A; N/A; European	None	166.0	151.0	<0.05
Soares (2010)^15^	Brazil	25/12	55.2 (7.8) for cases, 51.9 (4.3) for controls; 100%; Latin American	None	108.8	37.6	<0.05
Al-Hamodi (2011)^7^	Malaysia	303/131	51.0 (8.1) for cases, 47.2 (14.0) for controls; N/A; Asian	Gender, age, race	25.4	30.2	0.01
Mertens (2001)^110^	Belgium	30/30	63.0 (7.0) for cases, 62.0 (7.0) for controls; 100%; European	Age, weight, BMI, percent fat mass, total abdominal fat mass	*20.4*	*14.4*	0.004
Verkleij (2011)^14^	Netherlands	207/100	66 (10) for cases, 65 (10) for controls; 45%; European	Gender, age, smoking, calcium medication, antihypertensive medication	98	57	0.038
Zhong (2012)^111^	China	123/151	57.6 (8.0) for cases, 53.1 (7.3) for controls; 59.5%; Asian	None	6.2^b^	2.0^b^	<0.01

^a^PAI-1 measured as activity level (IU/mL, U/mL, or AU/mL) is indicated in italics.

^b^pmol/dL.

**Table 2 t2:** Baseline characteristics of prospective studies of plasminogen activator inhibitor-1 and incident type 2 diabetes.

Study	Study design/Follow-up, y	Country	Mean age (SD); % Women; Ethnicity	No. of cases/controls	Case ascertainment	Assay method	Adjustment
Festa *et al.* (2002)^9^	Prospective cohort/5.2	U.S.A.	56.0 (7.8) for cases, 54.6 (8.5) for controls; 43.5%; White, black, Hispanic	144/903	A standard 75-g OGTT was performed, and glucose tolerance status was based on the World Health Organization criteria	Citrated plasma using a two-site immunoassay	Age, sex, clinical center, smoking, ethnicity, S_I_, BMI, family history of diabetes, physical activity
Eliasson *et al.* (2003)^10^	Prospective cohort/9	Sweden	51.9 (8.7) for cases, 44.9 (10.9) for controls; 40.3%; European	15/ 536	Fasting glucose ≥7.0 mmol/L and/or post load glucose ≥11.1 mmol/L or self-report of diabetes diagnosis	Chromogenic assay	Age, sex, waist, DBP, fasting insulin, triglycerides
Kanaya *et al.* (2006)^12^	Prospective cohort/5	U.S.A.	73.0 (3.0) for cases, 74.0 (3.0) for controls; 53.4%; 38.4% black, 61.6% white	143/ 2213	Self-report of a new diabetes diagnosis, use of a diabetes medication, or fasting glucose ≥ 126 mg/dL	Citrated plasma samples using a 2-site ELISA	Age, sex, race, BMI, visceral fat, fasting glucose, fasting insulin, HDL cholesterol, triglycerides, hypertension, leptin, adiponectin
Davidson *et al.* (2006)^24^	Prospective cohort/4	U.S.A.	N/A; N/A; American Indian	137/ 1079	Treatment with insulin or oral glucose-lowering agents, or fasting glucose ≥7.0 mmol/L	Immunoassay	Age, sex, study center, waist, CRP, fibrinogen, triglyceride, SBP, insulin
Meigs *et al.* (2006)^23^	Prospective cohort/7	U.S.A.	54 (10.9); 54.4%; Primarily white	153/ 2771	Fasting plasma glucose level ≥7.0 mmol/l or use of hypoglycemic drug therapy	ELISA-method	^a^Sex, physical activity, HDL cholesterol, triglycerides, smoking, parental history of diabetes, BP, IFG/IGT, use of exogenous estrogen, alcohol, aspirin or NSAIDs, BP therapy, WC, HOMA-IR, CRP
Stranges *et al.* (2008)^27^	Nested case-control/5.9	U.S.A.	58.13 (10.59) for cases, 59.83 (10.48) for controls; 47.5%; Mainly white	54/ 151	Diagnosed by their physician and taking antidiabetic medications, or fasting glucose > 125 mg/dl	Two-site ELISA	Age, gender, race/ ethnicity, year of baseline visit, baseline fasting glucose (<110 or 110–125 mg/dL)
Alessi *et al.* (2011)^21^	Nested Case-control/9	France	50.6 (9.0) for cases, 50.6 (8.9) for controls; N/A; European	182/ 363	Fasting glucose ≥7.0 mmol/L or self-reported taking drugs for diabetes	EDTA plasma, using an immune-reactivity assay.	Age, sex, insulin, CRP, BMI, vitronectin
Hernestal-Boman *et al.* (2012)^22^	Nested case-control/5.5	Sweden	50.5 (8.1) for cases, 50.2 (8.3) for controls; 43.3% European	152/ 260	Diabetic patients were defined by FPG and OGTT according to World Health Organisation criteria 1999 or self-report of diagnosis	ELISA-assay	Age, sex, year of health exam, BMI, smoking, family history of T2D, physical activity, CRP, SBP, triglycerides, fasting glucose, 2 hour capillary glucose

SBP, systolic blood pressure. DBP, diastolic blood pressure. NSAID, nonsteroidal anti-inflammatory drug. S_I_, insulin sensitivity index. vWF, von Willebrand factor. FPG, fasting plasma glucose. ^a^For sub-group analyses by baseline glucose tolerance status (Fig. 3), Meigs *et al.* adjusted for the following covariates only: age, sex, physical activity, HDL cholesterol and triglyceride level, smoking, parental history of diabetes, blood pressure level, IFG/IGT, and use of exogenous estrogen, alcohol, aspirin or nonsteroidal anti-inflammatory drugs, and blood pressure therapy

**Table 3:** Newcastle-Ottawa Quality Assessment Scale – Cohort Studies.

	Representativeness of the exposed cohort	Selection	Ascertainment of exposure	Demonstration that outcome of interest was not present at start of study	Comparability	Assessment of outcome	Outcome	Adequacy of follow up of cohorts	Total
		Selection of the non-exposed cohort			Comparability of cohorts on the basis of the design or analysis (study adjusts for age*, sex*)		Was follow-up long enough for outcomes to occur		
Festa *et al.* (2002)^9^	*	*	*	*	**	*	*	—	8
Eliasson *et al.* (2003)^10^	*	*	*	*	**	*	*	—	8
Kanaya *et al.* (2006)^12^	*	*	*	*	**	*	*	*	9
Davidson *et al.* (2006)^24^	—	*	*	*	**	*	—	—	6
Meigs *et al.* (2006)^23^	*	*	*	*	**	*	*	—	8

**Table 4:** Newcastle-Ottawa Quality Assessment Scale – Case-control studies.

	Is the case definition adequate?	Selection	Selection of Controls	Definition of Controls	Comparability	Ascertainment of exposure	Outcome	Non-response rate	Score
		Representativeness of the cases			Comparability of cases and controls on the basis of the design or analysis (study adjusts for age*, sex*)		Same method of ascertainment for cases and controls		
Stranges *et al.* (2008)^27^	*	*	*	*	**	*	*	*	9
Alessi *et al.* (2011)^21^	*	*	*	*	**	*	*	*	9
Hernestal-Boman *et al.* (2012)^22^	*	*	*	*	**	*	*	—	8

## References

[b1] Grant P.J. Diabetes mellitus as a prothrombotic condition. J Intern Med. 262, 157–72 (2007). 17645584 10.1111/j.1365-2796.2007.01824.x

[b2] Schäfer K., Fujisawa K., Konstantinides S. & Loskutoff D.J. Disruption of the plasminogen activator inhibitor 1 gene reduces the adiposity and improves the metabolic profile of genetically obese and diabetic ob/ob mice. FASEB J. 15, 1840–2 (2001). 11481248 10.1096/fj.00-0750fje

[b3] Ma L.J. *et al.* Prevention of obesity and insulin resistance in mice lacking plasminogen activator inhibitor 1. Diabetes 53, 336–46 (2004). 14747283 10.2337/diabetes.53.2.336

[b4] Festa A. *et al.* Relative contribution of insulin and its precursors to fibrinogen and PAI-1 in a large population with different states of glucose tolerance. The Insulin Resistance Atherosclerosis Study (IRAS). Arterioscler Thromb Vasc Biol. 19, 562–8 (1999). 10073958 10.1161/01.atv.19.3.562

[b5] Alessi M.C. *et al.* Production of plasminogen activator inhibitor 1 by human adipose tissue: possible link between visceral fat accumulation and vascular disease. Diabetes. 46, 860–7 (1997). 9133556 10.2337/diab.46.5.860

[b6] Meigs J.B. *et al.* Hyperinsulinemia, hyperglycemia, and impaired hemostasis: The Framingham Offspring Study. JAMA 283, 221–8 (2000). 10634338 10.1001/jama.283.2.221

[b7] Al-Hamodi Z., Ismail I.S., Saif-Ali R., Ahmed K.A. & Muniandy S. Association of plasminogen activator inhibitor-1 and tissue plasminogen activator with type 2 diabetes and metabolic syndrome in Malaysian subjects. Cardiovasc Diabetol. 10, 23 (2011). 21414238 10.1186/1475-2840-10-23 PMC3064636

[b8] Nagi D.K., Ali V.M., Jain S.K., Walji S. & Yudkin J.S. Plasminogen Activator inhibitor (PAI-1) activity is elevated in asian and caucasian subjects with non-insulin-dependent (Type 2) Diabetes but not in those with impaired glucose tolerance (IGT) or non-diabetic Asians. Diabet Med. 13, 59–64 (1996). 8741814 10.1002/(SICI)1096-9136(199601)13:1<59::AID-DIA2>3.0.CO;2-Z

[b9] Festa A., D’Agostino R., Tracy R.P. & Haffner S.M. Insulin Resistance Atherosclerosis Study. Elevated levels of acute-phase proteins and plasminogen activator inhibitor-1 predict the development of type 2 diabetes: The Insulin Resistance Atherosclerosis Study. Diabetes. 51, 1131–7 (2002). 11916936 10.2337/diabetes.51.4.1131

[b10] Eliasson M.C., Jansson J.H., Lindahl B. & Stegmayr B. High levels of tissue plasminogen activator (tPA) antigen precede the development of type 2 diabetes in a longitudinal population study. The Northern Sweden MONICA Study. Cardiovasc Diabetol. 2, 19 (2003). 14690546 10.1186/1475-2840-2-19 PMC328088

[b11] Kanaya A.M. *et al.* Adipocytokines attenuate the association between visceral adiposity and diabetes in older adults. Diabetes Care. 27, 1375–80 (2004). 15161791 10.2337/diacare.27.6.1375

[b12] Kanaya A.M. *et al.* Adipocytokines and incident diabetes mellitus in older adults: the independent effect of plasminogen activator inhibitor 1. Arch Intern Med. 166, 350–6 (2006). 16476877 10.1001/archinte.166.3.350

[b13] Verkleij C.J. *et al.* The hemostatic system in patients with type 2 diabetes with and without cardiovascular disease. Clin Appl Thromb Hemost. 17, E57–63 (2011). 21078616 10.1177/1076029610384112

[b14] Soares A.L. *et al.* PAI-1 and D-dimer in type 2 diabetic women with asymptomatic macrovascular disease assessed by carotid Doppler. Clin Appl Thromb Hemost. 16, 204–8 (2010). 19825919 10.1177/1076029609334626

[b15] Kitagawa N. *et al.* Different metabolic correlations of thrombin-activatable fibrinolysis inhibitor and plasminogen activator inhibitor-1 in non-obese type 2 diabetic patients. Diabetes Res Clin Pract. 73, 150–7 (2006). 16458385 10.1016/j.diabres.2005.12.008

[b16] Hughes K., Choo M., Kuperan P., Ong C.N. & Aw T.C. Cardiovascular risk factors in non-insulin-dependent diabetics compared to non-diabetic controls: a population-based survey among Asians in Singapore. Atherosclerosis. 136, 25–31 (1998). 9544728 10.1016/s0021-9150(97)00180-9

[b17] DeClerck PJ., Alessi MC., Verstreken M., Kruithof EK., Juhan-Vague I. & Collen D. Measurement of plasminogen activator inhibitor 1 in biologic fluids with a murine monoclonal antibody-based enzyme-linked immunosorbent assay. Blood. 71, 220–5 (1988). 3257145

[b18] Stroup D.F. *et al.* Meta-analysis of observational studies in epidemiology: a proposal for reporting. Meta-analysis Of Observational Studies in Epidemiology (MOOSE) group. JAMA 283, 2008–12 (2000). 10789670 10.1001/jama.283.15.2008

[b19] Wells G. *et al.* *The Newcastle-Ottawa Scale (NOS) for assessing the quality of nonrandomised studies in meta-analyses*. (2011) Available at: http://www.ohri.ca/programs/clinical_epidemiology/oxford.asp. (Accessed 22 October 2014).

[b20] Danesh J., Collins R., Appleby P. & Peto R. Association of fibrinogen, C-reactive protein, albumin, or leukocyte count with coronary heart disease: meta-analyses of prospective studies. JAMA 279, 1477–82 (1998). 9600484 10.1001/jama.279.18.1477

[b21] Alessi M.C. *et al.* Association of vitronectin and plasminogen activator inhibitor-1 levels with the risk of metabolic syndrome and type 2 diabetes mellitus. Results from the D.E.S.I.R. prospective cohort. Thromb Haemost. 106, 416–22 (2011). 21800006 10.1160/TH11-03-0179

[b22] Hernestal-Boman J. *et al.* Signs of dysregulated fibrinolysis precede the development of type 2 diabetes mellitus in a population-based study. Cardiovasc Diabetol. 11, 152 (2012). 23249721 10.1186/1475-2840-11-152 PMC3538597

[b23] Meigs J.B. *et al.* Hemostatic markers of endothelial dysfunction and risk of incident type 2 diabetes The Framingham Offspring Study. Diabetes. 55, 530–7 (2006). 16443791 10.2337/diabetes.55.02.06.db05-1041

[b24] Davidson M. *et al.* Plasminogen activator inhibitor-1 and the risk of Type 2 diabetes mellitus in American Indians: the Strong Heart Study. Diabet Med. 23, 1158–9 (2006). 16978385 10.1111/j.1464-5491.2006.01923.x

[b25] Begg C.B. & Mazumdar M. Operating characteristics of a rank correlation test for publication bias. Biometrics 50, 1088–101 (1994). 7786990

[b26] Egger M., Davey Smith G., Schneider M. & Minder C. Bias in meta-analysis detected by a simple, graphical test. BMJ 315, 629–34 (1997). 9310563 10.1136/bmj.315.7109.629 PMC2127453

[b27] Stranges S. *et al.* Additional contribution of emerging risk factors to the prediction of the risk of type 2 diabetes: evidence from the Western New York Study. Obesity. 16, 1370–6 (2008). 18356828 10.1038/oby.2008.59

[b28] Raji M.A., Al Snih S., Ray L.A., Patel K.V. & Markides K.S. Cognitive status and incident disability in older Mexican Americans: findings from the Hispanic established population for the epidemiological study of the elderly. Ethn Dis. 14, 26–31 (2004). 15002920

[b29] Lutsey P.L. *et al.* Plasma hemostatic factors and endothelial markers in four racial/ethnic groups: the MESA study. J Thromb Haemost. 4, 2629–35 (2006). 17002663 10.1111/j.1538-7836.2006.02237.x

[b30] Festa A. *et al.* Promoter (4G/5G) plasminogen activator inhibitor-1 genotype and plasminogen activator inhibitor-1 levels in blacks, Hispanics, and non-Hispanic whites: The Insulin Resistance Atherosclerosis Study. Circulation. 107, 2422–7 (2003). 12719278 10.1161/01.CIR.0000066908.82782.3A

[b31] Oldroyd J., Banerjee M., Heald A. & Cruickshank K. Diabetes and ethnic minorities. Postgrad Med J. 81, 486–90 (2005). 16085737 10.1136/pgmj.2004.029124 PMC1743339

[b32] Palaniappan L.P., Carnethon M.R. & Fortmann S.P. Heterogeneity in the relationship between ethnicity, BMI, and fasting insulin. Diabetes Care 25, 1351–7 (2002). 12145234 10.2337/diacare.25.8.1351 PMC3121929

[b33] Schmidt M.I. *et al.* Markers of inflammation and prediction of diabetes mellitus in adults (Atherosclerosis Risk in Communities study): a cohort study. Lancet 353, 1649–52 (1999). 10335783 10.1016/s0140-6736(99)01046-6

[b34] Duncan B.B. *et al.* Factor VIII and other hemostasis variables are related to incident diabetes in adults. Diabetes Care 22, 767–72 (1999). 10332679 10.2337/diacare.22.5.767

[b35] Wannamethee S.G. *et al.* Tissue plasminogen activator, von Willebrand factor, and risk of type 2 diabetes in older men. Diabetes Care 31, 995–1000 (2008). 18235054 10.2337/dc07-1569

[b36] Thorand B. *et al.* Elevated markers of endothelial dysfunction predict type 2 diabetes mellitus in middle-aged men and women from the general population. Arterioscler Thromb Vasc Biol. 26, 398–405 (2006). 16322530 10.1161/01.ATV.0000198392.05307.aa

[b37] Song Y. *et al.* Circulating levels of endothelial adhesion molecules and risk of diabetes in an ethnically diverse cohort of women. Diabetes 56, 1898–904 (2007). 17389327 10.2337/db07-0250 PMC1952236

[b38] Lowe G.D.O., Rumley A., Whincup P.H. & Danesh J. Hemostatic and rheological variables and risk of cardiovascular disease. Semin Vasc Med. 2, 429–39 (2002). 16222632 10.1055/s-2002-36771

[b39] Festa A., Williams K., Tracy R.P., Wagenknecht L.E. & Haffner S.M. Progression of plasminogen activator inhibitor-1 and fibrinogen levels in relation to incident type 2 diabetes. Circulation. 113, 1753–9 (2006). 16585388 10.1161/CIRCULATIONAHA.106.616177

[b40] Huang J. *et al.* Genome-wide association study for circulating levels of PAI-1 provides novel insights into its regulation. Blood. 120, 4873–81 (2012). 22990020 10.1182/blood-2012-06-436188 PMC3520624

[b41] Lee K.W. & Lip G.Y.H. Effects of lifestyle on hemostasis, fibrinolysis, and platelet reactivity. Arch Intern Med. 163, 2368–92 (2003). 14581258 10.1001/archinte.163.19.2368

[b42] Nagi D.K. & Yudkin J.S. Effects of metformin on insulin resistance, risk factors for cardiovascular disease, and plasminogen activator inhibitor in NIDDM subjects. Diabetes Care 16, 621–29 (1993). 8462390 10.2337/diacare.16.4.621

[b43] Fonseca V.A. *et al.* Effect of troglitazone on fibrinolysis and activated coagulation in patients with non–insulin-dependent diabetes mellitus. J Diabetes Complications 12, 181–86 (1998). 9647334 10.1016/s1056-8727(97)00109-8

[b44] Ingelsson E. *et al.* Multimarker approach to evaluate the incidence of the metabolic syndrome and longitudinal changes in metabolic risk factors. Circulation. 116, 984–92 (2007). 17698726 10.1161/CIRCULATIONAHA.107.708537

[b45] Mertens I. & Van Gaal L.F. Obesity, haemostasis and the fibrinolytic system. Obes Rev. 3, 85–101 (2002). 12120424 10.1046/j.1467-789x.2002.00056.x

[b46] Shimomura I. *et al.* Enhanced expression of PAI-1 in visceral fat: possible contributor to vascular disease in obesity. Nat Med. 2, 800–3 (1996). 8673927 10.1038/nm0796-800

[b47] Alessi M.C. *et al.* Production of plasminogen activator inhibitor 1 by human adipose tissue: possible link between visceral fat accumulation and vascular disease. Diabetes. 46, 860–7 (1997). 9133556 10.2337/diab.46.5.860

[b48] Halleux C.M., Declerck P.J., Tran S.L., Detry R. & Brichard S.M. Hormonal control of plasminogen activator inhibitor-1 gene expression and production in human adipose tissue: stimulation by glucocorticoids and inhibition by catecholamines. J Clin Endocrinol Metab. 84, 4097–105 (1999). 10566656 10.1210/jcem.84.11.6127

[b49] Landin K. *et al.* Abdominal obesity is associated with an impaired fibrinolytic activity and elevated plasminogen activator inhibitor-1. Metabolism. 39, 1044–8 (1990). 2215252 10.1016/0026-0495(90)90164-8

[b50] Janand-Delenne B. *et al.* Visceral fat as a main determinant of plasminogen activator inhibitor 1 level in women. Int J Obes Relat Metab Disord. 22, 312–7 (1998). 9578235 10.1038/sj.ijo.0800585

[b51] Giltay E.J. *et al.* Visceral fat accumulation is an important determinant of PAI-1 levels in young, nonobese men and women: modulation by cross-sex hormone administration. Arterioscler Thromb Vasc Biol. 18, 1716–22 (1998). 9812909 10.1161/01.atv.18.11.1716

[b52] Marckmann P., Toubro S. & Astrup A. Sustained improvement in blood lipids, coagulation, and fibrinolysis after major weight loss in obese subjects. Eur J Clin Nutr. 52, 329–333 (1998). 9630382 10.1038/sj.ejcn.1600558

[b53] Aziz CB. *et al.* Reduced fibrinogen, fibrinolytic biomarkers, and physical parameters after a weight-loss program in obese subjects. N Am J Med Sci. 6, 377–82 (2014). 25210670 10.4103/1947-2714.139286 PMC4158645

[b54] Folsom A.R. *et al.* Impact of weight loss on plasminogen activator inhibitor (PAI-1), factor VII, and other hemostatic factors in moderately overweight adults. Arterioscler Thromb Vasc Biol. 13, 162–9 (1993). 10.1161/01.atv.13.2.162 8427853

[b55] Mavri A. *et al.* Impact of adipose tissue on plasma plasminogen activator inhibitor-1 in dieting obese women. Arterioscler Thromb Vasc Biol. 19, 1582–7 (1999). 10364094 10.1161/01.atv.19.6.1582

[b56] Dandona P., Aljada A. & Bandyopadhyay A. Inflammation: the link between insulin resistance, obesity and diabetes. Trends Immunol. 25, 4–7 (2004). 14698276 10.1016/j.it.2003.10.013

[b57] Devaraj S., Xu D.Y. & Jialal I. C-reactive protein increases plasminogen activator inhibitor-1 expression and activity in human aortic endothelial cells: implications for the metabolic syndrome and atherothrombosis. Circulation. 107, 398–404 (2003). 12551862 10.1161/01.cir.0000052617.91920.fd

[b58] Alessi M.C. *et al.* Plasminogen activator inhibitor 1, transforming growth factor-beta1, and BMI are closely associated in human adipose tissue during morbid obesity. Diabetes. 49, 1374–80 (2000). 10923640 10.2337/diabetes.49.8.1374

[b59] Cigolini M. *et al.* Expression of plasminogen activator inhibitor-1 in human adipose tissue: a role for TNF-alpha? Atherosclerosis 143, 81–90 (1999). 10208482 10.1016/s0021-9150(98)00281-0

[b60] Birgel M., Gottschling-Zeller H., Röhrig K. & Hauner H. Role of cytokines in the regulation of plasminogen activator inhibitor-1 expression and secretion in newly differentiated subcutaneous human adipocytes. Arterioscler Thromb Vasc Biol. 20, 1682–7 (2000). 10845889 10.1161/01.atv.20.6.1682

[b61] Singh P. *et al.* Leptin upregulates the expression of plasminogen activator inhibitor-1 in human vascular endothelial cells. Biochem Biophys Res Commun. 392, 47–52 (2010). 20051227 10.1016/j.bbrc.2009.12.158 PMC2831408

[b62] Komiya M. *et al.* Bi-directional regulation between adiponectin and plasminogen activator-inhibitor-1 in 3T3-L1 cells. In Vivo. 28, 13–9 (2014). 24425831

[b63] Chen G.C., Qin L.Q. & Ye J.K. Leptin levels and risk of type 2 diabetes: gender-specific meta-analysis. Obes Rev. 15, 134–42 (2014). 24102863 10.1111/obr.12088

[b64] Li S., Shin H.J., Ding E.L. & van Dam R.M. Adiponectin levels and risk of type 2 diabetes: a systematic review and meta-analysis. JAMA. 302, 179–88 (2009). 19584347 10.1001/jama.2009.976

[b65] Samad F., Pandey M., Bell P.A. & Loskutoff D.J. Insulin continues to induce plasminogen activator inhibitor 1 gene expression in insulin-resistant mice and adipocytes. Mol Med. 6, 680–92 (2000). 11055587 PMC1949975

[b66] Bastard J.P. & Pieroni L. Plasma plasminogen activator inhibitor 1, insulin resistance and android obesity. Biomed Pharmacother. 53, 455–61 (1999). 10665338 10.1016/s0753-3322(00)88103-2

[b67] Sjoholm A. & Nystrom T. Endothelial inflammation in insulin resistance. Lancet. 365, 610–2 (2005). 15708106 10.1016/S0140-6736(05)17912-4

[b68] Pandolfi A. *et al.* Glucose and insulin independently reduce the fibrinolytic potential of human vascular smooth muscle cells in culture. Diabetologia. 39, 1425–31 (1996). 8960822 10.1007/s001250050594

[b69] Suzuki M., Akimoto K. & Hattori Y. Glucose upregulates plasminogen activator inhibitor-1 gene expression in vascular smooth muscle cells. Life Sci. 72, 59–66 (2002). 12409145 10.1016/s0024-3205(02)02182-3

[b70] Gabriely I. *et al.* Hyperglycemia induces PAI-1 gene expression in adipose tissue by activation of the hexosamine biosynthetic pathway. Atherosclerosis 160, 115–22 (2002). 11755928 10.1016/s0021-9150(01)00574-3

[b71] Maiello M. *et al.* Increased expression of tissue plasminogen activator and its inhibitor and reduced fibrinolytic potential of human endothelial cells cultured in elevated glucose. Diabetes 41, 1009–15 (1992). 1628760 10.2337/diab.41.8.1009

[b72] Lee C.C. *et al.* The association of inflammatory and fibrinolytic proteins with 5 year change in insulin clearance: the Insulin Resistance Atherosclerosis Study (IRAS). Diabetologia 56, 112–20 (2013). 10.1007/s00125-012-2741-8 PMC4010386 23052060

[b73] Azad N. *et al.* Association of PAI-1 and fibrinogen with diabetic retinopathy in the Veterans Affairs Diabetes Trial (VADT). Diabetes Care 37, 501–6 (2014). 24101699 10.2337/dc13-1193 PMC3898766

[b74] Nicholas SB. *et al.* Plasminogen activator inhibitor-1 deficiency retards diabetic nephropathy. Kidney Int. 67, 1297–307 (2005). 15780082 10.1111/j.1523-1755.2005.00207.x

[b75] Zhao L. & Huang P. Plasminogen activator inhibitor-1 4G/5G polymorphism is associated with type 2 diabetes risk. Int J Clin Exp Med. 6, 632–40 (2013). 24040470 PMC3762617

[b76] Auwerx J., Bouillon R., Collen D. & Geboers J. Tissue-type plasminogen activator antigen and plasminogen activator inhibitor in diabetes mellitus. Arteriosclerosis 8, 68–72 (1988). 2449156 10.1161/01.atv.8.1.68

[b77] Juhan-Vague I. *et al.* Increased plasminogen activator inhibitor activity in non insulin dependent diabetic patients-relationship with plasma insulin. Thromb Haemost. 61, 370–3 (1989). 2678583

[b78] Rydzewski A., Kawamura H., Watanabe I., Takada Y. & Takada A. Plasminogen activators and plasminogen activator inhibitor (PAI-1) in type II diabetes mellitus. Fibrinolysis 4, 183–8 (1990).

[b79] Cho Y.W., *et al.* Plasma t-PA and PAI-1 antigen concentrations in non-insulin dependent diabetic patients: effects of treatment modality on fibrinolysis. Korean J Intern Med. 7, 81–6 (1992). 1306076 10.3904/kjim.1992.7.2.81 PMC4532107

[b80] Potter van Loon B.J., Kluft C., Radder J.K., Blankenstein M.A. & Meinders A.E. The cardiovascular risk factor plasminogen activator inhibitor type 1 is related to insulin resistance. Metabolism 8, 945–9 (1993). 8345817 10.1016/0026-0495(93)90005-9

[b81] Takada Y., Urano T., Watanabe I., Taminato A., Yoshimi T. & Takada A. Changes in fibrinolytic parameters in male patients with type 2 (non-insulin-dependent) diabetes mellitus. Thromb Res. 781, 405–15 (1993). 8236167 10.1016/0049-3848(93)90165-k

[b82] Avellone G., Di Garbo V. & Cordova R. Blood coagulation and fibrinolysis in obese NIDDM patients. Diabetes Res. 25, 85–92 (1994). 7648783

[b83] Park Y.S., *et al.* The effect of obesity on fibrinolytic activity and plasma lipoprotein (a) levels in patients with type 2 diabetes mellitus in Korea. Diabetes Res Clin Pract. 24, 25–31 (1994). 7924883 10.1016/0168-8227(94)90082-5

[b84] Kario K. *et al.* Activation of tissue factor-induced coagulation and endothelial cell dysfunction in non–insulin-dependent diabetic patients with microalbuminuria. Arterioscler Thromb Vasc Biol. 15, 1114–20 (1995). 7627704 10.1161/01.atv.15.8.1114

[b85] Ito Y., Okeda T., Sato Y., Ito M. & Sakata T. Plasminogen activator inhibitor-1 in nonobese subjects with non-insulin-dependent diabetes mellitus. Proc Soc Exp Biol Med. 211, 287–91 (1996). 8633110 10.3181/00379727-211-43973

[b86] Akanji A.O., Abdullah A. & Tahzeeb S. Lipoprotein(a), tissue plasminogen activator and plasminogen activator inhibitor 1 levels in hyperlipidaemic patients in Kuwait. Eur J Clin Invest. 27, 380–6 (1997). 9179544 10.1046/j.1365-2362.1997.1230671.x

[b87] Gray R.P., Panahloo A., Mohamed-Ali V., Patterson D.L.H. & Yudkin J.S. Proinsulin-like molecules and plasminogen activator inhibitor type 1 (PAI-1) activity in diabetic and non-diabetic subjects with and without myocardial infarction. Atherosclerosis 130,171–8 (1997). 9126661 10.1016/s0021-9150(96)06070-4

[b88] Krekora K. *et al.* Decrease in urokinase-type plasminogen activator (u-PA) levels in patients with non-insulin dependent diabetes mellitus. Fibrinolysis & Proteolysis 4, 215–9 (1997).

[b89] Bannan S., Mansfield M.W. & Grant P.J. Soluble vascular cell adhesion molecule-1 and E-selectin levels in relation to vascular risk factors and to E-selectin genotype in the first degree relatives of NIDDM patients and in NIDDM patients. Diabetologia 41, 460–6 (1998). 9562351 10.1007/s001250050930

[b90] Temelkova-Kurktschiev T., Siegert G., Koehler C., Henkel E. & Hanefeld E. Impaired fibrinolysis and early atherosclerosis in impaired glucose tolerance and newly detected type 2 diabetes. Fibrinolysis & Proteolysis 13, 11–5 (1999).

[b91] Testa R. *et al.* A significant relationship between plasminogen activator inhibitor type-1 and lipoprotein(a) in non-insulin-dependent diabetes mellitus without complications. Int J Clin Lab Res. 28, 187–191 (1998). 9801931 10.1007/s005990050042

[b92] Testa R. *et al.* A strong inverse relationship between PAI-1 and Lp(a) in hypertensive Type 2 diabetic patients. Diab Nutr Metab. 12, 400–6 (1999). 10782561

[b93] Hernandez C., Chacon P., Garcia-Pascual L., Mesa J. & Simo R. Relationship between lipoprotein(a) phenotypes and plasminogen activator inhibitor type 1 in diabetic patients. Thromb Res. 15, 119–27 (2000). 10946085 10.1016/s0049-3848(00)00248-6

[b94] Testa R. *et al.* Fibronectin and lipoprotein(a) are inversely related to plasminogen activator inhibitor type-1 levels in Type 2 diabetic patients without complications. Diab. Nutr. Metab. 13, 269–275 (2000). 11105969

[b95] Zareba W. *et al.* Increased level of von Willebrand factor is significantly and independently associated with diabetes in postinfarction patients. Thromb Haemost. 86, 791–9 (2001). 11583309

[b96] Aso Y. *et al.* Impaired fibrinolytic compensation for hypercoagulability in obese patients with type 2 diabetes: association with increased plasminogen activator inhibitor-1. Metabolism. 51, 471–6 (2002). 11912556 10.1053/meta.2002.31334

[b97] Brandenburg S.L. *et al.* Impaired fibrinolysis in premenopausal women and age-matched men with Type 2 diabetes mellitus: a pilot study. J Investig Med. 50, 110–5 (2002). 10.2310/6650.2002.31292 11930948

[b98] Leurs P.B. *et al.* Tissue factor pathway inhibitor and other endothelium-dependent hemostatic factors in elderly individuals with normal or impaired glucose tolerance and type 2 diabetes. Diabetes Care 25, 1340–5 (2002). 12145232 10.2337/diacare.25.8.1340

[b99] Fattah M.A., Shaheen M.H. & Mahfouz M.H. Disturbances of haemostasis in diabetes mellitus. Dis Markers. 19, 251–8 (2004). 15258325 10.1155/2004/797458 PMC3850632

[b100] Yu Y., Suo L., Yu H., Wang C. & Tang H. Insulin resistance and endothelial dysfunction in type 2 diabetes patients with or without microalbuminuria. Diabetes Res Clin Pract. 65, 95–104 (2004). 15223221 10.1016/j.diabres.2004.01.006

[b101] Erem C. *et al.* Coagulation and fibrinolysis parameters in type 2 diabetic patients with and without diabetic vascular complications. Med Princ Pract. 14, 22–30 (2005). 15608477 10.1159/000081919

[b102] Soares A.L. *et al.* Type 2 diabetes: assessment of endothelial lesion and fibrinolytic system markers. Blood Coagul Fibrinolysis. 18, 395–9 (2007). 17581312 10.1097/MBC.0b013e328133f70f

[b103] Le D.S. *et al.* The association of plasma fibrinogen concentration with diabetic microvascular complications in young adults with early-onset of type 2 diabetes. Diabetes Res Clin Pract. 82, 317–23 (2008). 18922595 10.1016/j.diabres.2008.08.019

[b104] Romuk E. *et al.* Evaluation of VCAM-1 and PAI-1 concentration in diabetes mellitus patients. Diabet Dośw i Klin. 8, 85–8 (2008).

[b105] Sahli D., Eriksson J.W., Boman K. & Svensson M.K. Tissue plasminogen activator (tPA) activity is a novel and early marker of asymptomatic LEAD in type 2 diabetes. Thromb Res. 123, 701–6 (2009). 18945481 10.1016/j.thromres.2008.07.015

[b106] Jax T.W., Peters A.J., Plehn G. & Schoebel F.C. Relevance of hemostatic risk factors on coronary morphology in patients with diabetes mellitus type 2. Cardiovasc Diabetol. 8, 24 (2009). 19419582 10.1186/1475-2840-8-24 PMC2688504

[b107] Blaszkowski A., Galar M., Sokolowski J. & Kloczko J. Plasminogen activator inhibitor-1 and thrombin activable fibrinolysis inhibitor in patients with type 2 diabetes. Diabetologia 53 [Supp 1]: S525 (2010).

[b108] Kubisz P. *et al.* Circulating vascular endothelial growth factor in the normo- and/or microalbuminuric patients with type 2 diabetes mellitus. Acta Diabetologica. 47, 119–124 (2010). 19436948 10.1007/s00592-009-0127-2

[b109] Kovalyova O., Ambrosova T. & Shapovalova S. Diabetes is associated with hypoadiponectinema and elevated levels of TNF-alpha, PAI-1 in obese hypertensive patients. Journal of Diabetes 3 [Supplement S1]:45 (2011) (Abstract).

[b110] Mertens I. *et al.* Visceral fat is a determinant of PAI-1 activity in diabetic and non-diabetic overweight and obese women. Horm Metab Res. 33, 602–7 (2001). 11607880 10.1055/s-2001-17907

[b111] Zhong Z.L. & Chen S. Plasma plasminogen activator inhibitor-1 is associated with end-stage proliferative diabetic retinopathy in the Northern Chinese Han population. Exp Diabetes Res. 2012, 350852 (2012). 23304115 10.1155/2012/350852 PMC3518968

[b112] Brazionis L., Rowley K., Jenkins A., Itsiopoulos C. & O’Dea K. Plasminogen activator inhibitor-1 activity in type 2 diabetes: a different relationship with coronary heart disease and diabetic retinopathy. Arterioscler Thromb Vasc Biol. 28, 786–91 (2008). 18239151 10.1161/ATVBAHA.107.160168

