# Supplementary material for: Plasminogen activator inhibitor-1 and type 2 diabetes: a systematic review and meta-analysis of observational studies
Source: Sci Rep. 2016 Jan 27;6:17714. doi: 10.1038/srep17714 (PMC4728395; doi:10.1038/srep17714)
Supplement: Supplementary Information [file srep17714-s1.pdf]

Plasminogen activator inhibitor-1 and type 2 diabetes: a systematic review and meta-analysis of observational studies

James Yarmolinsky, Natália Bordin Barbieri, Tobias Weinmann, Patricia K. Ziegelmann, Bruce B. Duncan, Maria Inês Schmidt

Supplementary Data 1

EMBASE:

"Thrombotic factors" OR fibrinolysis OR "fibrinolytic agent" OR "fibrinolytic factor" OR hemostasis OR "hemostatic agent" OR "plasminogen activator inhibitor 1" OR "PAI 1 protein" AND "non insulin dependent diabetes"

PubMed:

(((((((plasminogen activator inhibitor 1[MeSH Terms]) OR Serpine1 protein, human[Supplementary Concept]) OR hemostasis[MeSH Terms]) OR fibrinolysis[MeSH Terms]) OR fibrinolytic agents[MeSH Terms]) OR thrombosis[MeSH Terms])) AND diabetes mellitus, type 2[MeSH Terms]

Web of Science:

TS=(thrombosis) OR TS=(thrombotic factor$) OR TS=(fibrinolysis) OR TS=(fibrinolytic agent$) OR TS=(fibrinolytic factor$) OR TS=(h$emostasis) OR TS=(h$emostatic factor$) OR TS=(plasminogen activator inhibitor type 1) OR TS=(Plasminogen activator inhibitor-1) OR TS=(Plasminogen activator inhibitor 1) OR TS=(PAI-1) OR TS=(PAI 1) OR TS=(Endothelial plasminogen activator inhibitor) OR TS=(serpin E1) AND [TS=(type 2 diabetes) OR TS=(type 2 diabetes mellitus) TS=(non-insulin-dependent diabetes) OR TS=(noninsulin-dependent diabetes) OR TS=(adult-onset diabetes) OR (TS=T2DM)].

Cochrane Library:

Plasminogen Activator Inhibitor 1 OR Hemostasis OR Fibrinolysis OR Fibrinolytic Agents OR Thrombosis OR Diabetes Mellitus, Type 2
